# Rotavirus within day care centres in Oxfordshire, UK: characterization of partial immunity

**DOI:** 10.1098/rsif.2008.0115

**Published:** 2008-05-13

**Authors:** L.J White, J Buttery, B Cooper, D.J Nokes, G.F Medley

**Affiliations:** 1 Mahidol Oxford Research Unit, Faculty of Tropical Medicine, Mahidol UniversityBangkok 10400, Thailand; 2 NHMRC Immunisation CCRE, Murdoch Childrens Research InstituteVic. 3052, Australia; 3 Department of Paediatrics, University of MelbourneVic. 3010, Australia; 4 Statistics, Modelling and Bioinformatics Department, Centre for Infections, Health Protection AgencyLondon NW9 5EQ, UK; 5 Department of Biological Sciences, University of WarwickCoventry CV4 7AL, UK; 6 Centre for Geographic Medicine Research—Coast, Kenya Medical Research InstitutePO Box 230, Kilifi, Kenya

**Keywords:** rotavirus, partial immunity, transmission dynamics, Markov chain Monte Carlo, DCC, day care centre, MCMC, Markov chain Monte Carlo, DIC, deviance information criterion

## Abstract

Repeated measures data for rotavirus infection in children within 14 day care centres (DCCs) in the Oxfordshire area, UK, are used to explore aspects of rotavirus transmission and immunity. A biologically realistic model for the transmission of infection is presented as a set of probability models suitable for application to the data. Two transition events are modelled separately: incidence and recovery. The complexity of the underlying mechanistic model is reflected in the choice of the fixed variables in the probability models. Parameter estimation was carried out using a Bayesian Markov chain Monte Carlo method. We use the parameter estimates obtained to build a profile of the natural history of rotavirus reinfection in an individual child. We infer that rotavirus transmission in children in DCCs is dependent on the DCC prevalence, with symptomatic infection of longer duration, but no more infectious per day of infectious period, than asymptomatic infection. There was evidence that a recent previous infection reduces the risk of disease and, to a lesser extent, reinfection, but not duration of infection. The results provide evidence that partial immunity to rotavirus infection develops over several time scales.

## 1. Introduction

Rotavirus infection is the single most important cause of infectious diarrhoea and death globally in infants and young children (Offit 1998). It is highly transmissible in developed and developing countries alike and virtually all children will have experienced at least one infection by the age of 2 years (Fischer *et al*. 2002) and one episode of rotavirus gastroenteritis by the age of 5 years (Parashar *et al*. 2003). Parashar *et al*. (2006) estimate that each year rotavirus infection is responsible for roughly 600 000 deaths globally.

Reinfection is common in children (Offit 1998; Fischer *et al*. 2002) and adults (Griffin *et al*. 2002; Anderson & Weber 2004). Intestinal secretory antibodies, whose responses are of short duration, are pivotal to protection against rotavirus reinfection (Franco & Greenberg 2001). This would suggest that (even strain specific) immunity against reinfection is short lived. There is, however, good evidence that repeated rotavirus infection cumulatively protects against infection and associated diarrhoeal disease (Velazquez *et al*. 1996). Furthermore, protection has been shown to correlate with serum IgA antibody titre (Velázquez *et al*. 2000), such that two infections (regardless of symptoms) produce a titre that protects completely against subsequent moderate-to-severe diarrhoea, less so against mild illness and least against asymptomatic infection; the duration of this effect is unknown.

At the individual level, the natural history of infection has yet to be adequately characterized for the purposes of predicting population-level transmission dynamics. Relative risk of reinfection (based on the history of previous infection) was estimated by Fischer *et al*. (2002) using a birth cohort, but their approach did not consider the transmission dynamics of the infection. Individual-level risk depends on population-level factors, such as infection prevalence and seasonal variation in contact rates, which interact with the natural history of infection at the individual level (e.g. duration of infection and immunity).

Here we combine population and individual levels using a generalized mechanistic mathematical model in conjunction with follow-up data. The model takes into account possible effects of an individual child's infection history on the risk of infection, duration of infection, infectiousness and severity of symptoms. This is performed within the dynamic context of the time-varying exposure levels arising from infection within day care centres (DCCs). Parameter estimates are obtained using a Markov chain Monte Carlo (MCMC) estimation method, which has the provision for variable intersample periods. This analysis is designed to establish the most likely natural history of infection and estimate the parameters connected with the resultant profile, thereby characterizing partial immunity.

## 2. Materials and methods

### 2.1 Rotavirus data

Two cohorts of children aged 6–24 months attending Oxfordshire DCCs were enrolled over consecutive rotavirus seasons in 1998–1999 and 1999–2000 (Buttery *et al*. submitted). DCCs were visited twice weekly and faecal samples collected if available in the previous 24 hours. The children were prospectively followed for episodes of diarrhoea or gastroenteritis, upon which additional stool samples were collected. The samples were assayed for rotavirus by reverse transcription and polymerase chain reaction detecting the rotaviral *VP1* (RNA polymerase) gene. One hundred and two children from 11 DCCs participated in the 1998–1999 season, and 80 children from 10 DCCs in 1999–2000.

There were 130 episodes of diarrhoea reported in 80 children. There were 68 episodes of rotavirus diarrhoea, and 244 episodes of asymptomatic carriage (ratio 1 : 3.6), representing a rate of 0.37 diarrhoeal and 1.34 asymptomatic infections per child season. The median interval between commencing DCC care and first episode of diarrhoea was 188 days (range 4–641). Most diarrhoeal episodes were mild, fewer than half of the responding families (41 out of 86, 47.6%) saw a primary care practitioner, and no child visited hospital for their illness. Fever was noted in only 17 out of 86 responses. All but four children had at least one positive rotavirus sample. Thirteen children had at least three separate episodes of infection. The raw data are in the form of 2948 sampling events from 185 children within 2 cohorts within 15 DCCs. We discounted one DCC since there were only three samples for just a single child. The data from the remaining 14 DCCs are represented on a timeline for each child (figure 1).

### 2.2 Model structure

We use a conceptual model similar to that developed and analysed in White & Medley (1998; figure 2). At any point in time, children are either uninfected or infected. Within the uninfected population there is a distribution of susceptibility to infection that is dependent on the time since recovery from previous infection. Note that we do not know the infection history of individuals from birth, thus we cannot explicitly model those never infected. The profile of susceptibility in the uninfected population changes over time, as a result of recruitment of individuals just recovered from an infection and their changing susceptibility to infection and disease over time since recovery.

The infected population comprises children with disease (an infection that involves an episode of diarrhoea is termed a symptomatic infection), and without (non-diarrhoeal infection). Upon infection there is some probability that the infection will involve symptoms, which can be dependent on the time between recovery from previous and current infections. The numbers in each of the infected categories change over time as a result of recruitment following infection of the uninfecteds.

The incidence per child per unit time is the (instantaneous) rate at which uninfected children become infected. The magnitude of this incidence is related to the proportion of infected children sharing the DCC. The model framework also allows for differential infectiousness between diseased and non-diseased children. The rate (per child per unit time) by which infected children move into the uninfected class (recovery) is assumed dependent on disease state, and also related to the time since previous infection. Uninfected individuals become infected at a rate dependent on the time elapsed since their previous infection (partial, temporary immunity) and the level and type (clinical versus subclinical) of infection to which they are exposed. We also include infection by sources outside of the DCC.

A family of models can be derived from this structure by making different assumptions about the dependence of susceptibility and recovery rate on disease state and/or time since previous infection. The model assumes all processes to be independent of child age, gender, the number of previous infections and the genotype of current and past infections. This mechanistic structure can be expressed in the form of a set of probability models. The number of previous infections was not measured in the study, since the children were not followed up from birth. We therefore use time since previous infection as a proxy for immunity level.

We therefore have a family of models for considering the mechanisms associated with each of incidence (models 1–5) and recovery (models 6–8). Model 1 does not include the effect of time since previous infection on susceptibility and only assumes that it is dependent on the nursery-level prevalence of infection regardless of symptoms. Model 1 is extended to model 2 by including different infectiousness for symptomatic versus asymptomatic infections. Model 1 is extended to model 3 by including the effect of time since previous infection on susceptibility. Model 1 is extended to model 4 by including a differential risk of symptomatic infection compared with asymptomatic infection based on the time since previous infection. Model 5 is a combination of models 4 and 3. Model 6 does not include the effect of time since previous infection or the existence of symptoms during the infection on duration of infection. Model 6 is extended to model 7 by including different durations of infection for symptomatic versus asymptomatic infections. Model 7 is extended to model 8 by including the effect of time since previous infection on duration of infection. The models are summarized in table 1.

The parameters were estimated using MCMC within WinBUGS v. 1.4.1 (Spiegelhalter *et al*. 2000). We assumed vague prior distributions for all parameters (i.e. a uniform distribution from zero to unity for parameters defined in that range and a uniform distribution from 0 to 1000 for the other parameters, since they were expected to have values much less than 1000; table 2).

### 2.3 Probability models

For sample *j* of child *i* in DCC *k*, *s*_*ijk*_=1 if the sample is positive and *s*_*ijk*_=0 if negative for rotavirus. For each sample, we recognize three measures of time: the number of weeks since the beginning of the study, *t*_*ijk*_; the days since the previous sample, *δt*_*ijk*_; and the weeks since the previous infection, *τ*_*ijk*_. The proportions of the DCC experiencing symptomatic and asymptomatic infections in each week are given by *y*_*d*_(*t*_*ijk*_,*k*) and *y*_*nd*_(*t*_*ijk*_,*k*), respectively. The transition events of incidence and recovery are denoted as *I*_*ijk*_ and *R*_*ijk*_, respectively. Table 3 summarizes the relationship between *s*_*ijk*_ and *s*_*i*(*j*−1)*k*_ and the transition indicator variables.

The function *m*_*ijk*_ allows us to estimate the risk of symptoms during an infection, *θ*(*τ*_*ijk*_),

(2.1) $$\begin{array}{ll} m_{ijk} & =\theta (\tau_{ijk})\;\text{if}\;\text{infected}\;\text{with}\;\text{symptoms},\\ & =1-\theta (\tau_{ijk})\;\text{if}\;\text{infected}\;\text{without}\;\text{symptoms},\\ & =1\;\text{if}\;\text{no}\;\text{infection}. \end{array}\}$$

The force of infection, *λ*_*ijk*_, acting on child *i* in DCC *k* on day *t*_*ijk*_ is the hazard of becoming infected. The force of infection also includes a constant non-DCC component, *λ*_0_. Then the force of infection can be constant, if *β*=0, (for all DCCs or for each DCC) or, if *β*>0, it can be dependent on the proportion of infected children with and without diarrhoea in the DCC. Children with diarrhoea and those without are equally infectious if *η*=0.5. If *η*>0.5, children with diarrhoea are more infectious than those without and are less if *η*<0.5.

(2.2) $$\lambda_{ijk}=\left\{2\beta \left[\eta y_{d}(t_{ijk},k)+(1-\eta )y_{nd}(t_{ijk},k)\right]+\lambda_{0}\right\}.$$

The recovery rate (the inverse of the average duration of infection, *δ*_*ijk*_) of child *i* at time *t*_*ijk*_ without the effect of previous infection is given by

(2.3) $$\begin{array}{ccc} \frac{1}{\delta_{ijk}}=\frac{2\rho }{\delta_{f}} & \;\text{if}\;\text{symptomatic},\\ =\frac{2(1-\rho )}{\delta_{f}} & \text{if}\;\text{asymptomatic}. & \end{array}\}$$

Children with diarrhoea and those without are assumed to have an equal duration of infection of *δ*_*f*_ if *ρ*=0.5. If *ρ*>0.5, children with diarrhoea recover more quickly than those without and more slowly if *ρ*<0.5.

We use a survival approach (Clayton & Hills 1998) to model the transition events of incidence and recovery. The transition probabilities for incidence, *I*_*ijk*_, and recovery, *R*_*ijk*_, are given by

(2.4) $$P\left(I_{ijk}=1|s_{i\left(j-1\right)k}=0\right)=m_{ijk}\left[1-\text{exp}\left(-f(\tau_{ijk})\lambda_{ijk}\delta t_{ijk}\right)\right],$$

and

(2.5) $$P\left(R_{ijk}=1|s_{i(j-1)k}=1\right)=1-\text{exp}\left(-\frac{\delta t_{ijk}}{g(\tau_{ijk})\delta_{ijk}}\right).$$

The function *m*_*ijk*_ of *θ* is included in equation (2.4) to allow us to differentiate between the incidence of symptomatic and asymptomatic infections. Thus, given that incidence has occurred, the probability that it is symptomatic is given by *θ*.

The incidence function refers only to samples from children with a previous negative sample, and thus the event of incidence (changing from negative to positive) is possible between the previous and current samples. Similarly, the recovery function refers only to samples from children with a previous positive sample.

The function *f*(*τ*_*ijk*_) allows us to explore the effect of the time elapsed since a previous infection, *τ*_*ijk*_, on the susceptibility of child *i* at time *t*_*ijk*_ to further infection. The function *g*(*τ*_*ijk*_) allows us to explore the effect of the time elapsed since a previous infection, *τ*_*ijk*_, on the duration of the current infection. A number of choices were considered for these functions and will be discussed in §2.4.

### 2.4 Data preparation

The time between samplings is variable (figure 3) and some intersample periods are too large to consider viable as repeated samples. That is, a child could have been reinfected a number of times during such periods. If we consider becoming infected as an event (*I*_*ijk*_), then the first sample (*j*=1) for child *k* is relevant only in that it informs the value of *I*_*i*2*k*_ for the second sample for the incidence dataset. Then all first sample values of *I*_*i*1*k*_ are unknown and not present in the final dataset. Also, all samples that occur too long after the last one (say Δ*t*_max_ days) are discarded. If Δ*t*_max_=21 days approximately 90% of the sampling intervals are included; and if Δ*t*_max_=14 days approximately 68% are included. The proportions *y*_*d*_(*t*_*ijk*_,*k*) and *y*_*nd*_(*t*_*ijk*_,*k*) were calculated before the samples were excluded.

If the intersample period is less than Δ*t*_max_, *I*_*ijk*_=1 if the current sample is positive and the prior sample negative, and *I*_*ijk*_=0 if the current and prior samples are negative. If the child was infected on their previous sample, then they are considered unexposed to infection and therefore the current value of *I*_*ijk*_ would be irrelevant and therefore not present in the dataset.

In order to consider the effect of low sensitivity (i.e. false negatives) of the test, we prepared two further datasets, *fn14-* and *fn7-data*, with the original dataset being *fn0-data*. In *fn14-data*, for a negative sample, if the previous and following samples are positive and are each within 14 days of the sample in question the sample was changed to positive. This occurred in 411 out of 2948 measurements. This was repeated with an interval of plus or minus 7 days to produce *fn7-data*. This occurred in 238 out of 2948 measurements. We report results in the text only for the *fn7-data* for clarity, but report results for all three datasets graphically. Table 4 gives the numbers of transitions for each dataset.

The primary aim of this study was to explore the effect of time elapsed since a previous infection on the three main characteristics—risk of infection, risk of disease and duration of infection—and therefore characterize partial immunity to rotavirus infection in the children participating in the study. In order to achieve this, only uninterrupted (i.e. each sample was within 14 days of the previous sample) data sequences occurring after their first measured infection were included for each child. Then, the time since previous infection could be measured. It was categorized into five categories ($0\leq \tau_{1}<2$, $2\leq \tau_{1}<4$, $4\leq \tau_{1}<6$, $6\leq \tau_{1}<12$ and $12\leq \tau_{1}<16$ weeks (there were no data for the final category for the recovery models)) to obtain profiles for each of the three characteristics.

### 2.5 Model selection and convergence

For each model, we computed the value of the deviance information criterion (DIC; Spiegelhalter *et al*. 2002). The model with the smallest DIC is the model that would best predict a replicate dataset of the same structure as that currently observed. We also plotted the distribution of the Pearson residuals (Green *et al*. 2004) to assess the validity of the models. A plot of the residuals can be obtained by arranging the predicted probabilities for each data point in ascending order with their corresponding Pearson residual, then separating them into equal sized groups and taking the sum of the residuals, *S*_R_, and the mean of the predicted probability, *μ*_P_. If *S*_R_ is plotted against *μ*_P_, the points should be randomly distributed around the *x*-axis.

Convergence was tested in each case using the Gelman–Rubin convergence diagnostic (Brooks & Gelman 1998) calculated within WinBUGS (Spiegelhalter *et al*. 2002). For each model, we ran three independent chains for 10 000 iterations following a burn in the period of 10 000 iterations (as this was sufficient to achieve convergence).

## 3. Results

The parameter estimates and the DIC of the nested models applied to the *fn7-data* are summarized in table 5.

Model 2 had a slightly higher DIC than model 1, also the confidence interval of the parameter *η* (altered infectiousness) included 0.5. Thus indicating that the inclusion of heterogeneous infectiousness based on symptoms does not improve the model fit. Model 6 had a higher DIC than model 7, also the confidence interval for parameter *ρ* (altered duration of infection) did not include 0.5. Thus indicating that the inclusion of heterogeneous duration of infection based on symptoms does improve the model fit.

Model 5 had a lower DIC than both models 3 and 4. Thus indicating that the inclusion of dependence of both susceptibility and risk of symptomatic infection on time since previous infection improves the model fit. The parameter *β*_*i*_ is the product of *β* and *f*(*τ*_*ijk*_) from equations (2.1) and (2.3). The parameter *θ*_*i*_ is *θ*(*τ*_*ijk*_) from equation (2.4). Except for *fn0-data*, when *βf*(*τ*_*ijk*_) is plotted against *τ*_*ijk*_, the risk of infection reaches a maximum at approximately 5 weeks since previous infection (figure 4*a*) and then falls. When *θ*(*τ*_*ijk*_) is plotted against *τ*_*ijk*_, the risk of symptoms given infection increases as time since infection increases (figure 4*b*).

Model 8 had a lower DIC than both models 6 and 7, thus indicating that the inclusion of dependence of duration of infection on time since previous infection improves the model fit. The parameter *δ*_*i*_ is the product of *δ*_*f*_ and *g*(*τ*_*ijk*_) from equations (2.2) and (2.5). When the duration of infection, *δ*_*f*_*g*(*τ*_*ijk*_), is plotted against *τ*_*ijk*_ it decreases as time since infection increases (figure 4*c*).

## 4. Discussion

The application of models to data from repeated infections of rotavirus within children within DCCs has resulted in the estimation of basic epidemiological parameters and the development of hypotheses on the protective effect of previous infection against infection and disease. The duration of infection is estimated at approximately 15 days and appears to be highly variable even for repeated infections within the same child. Symptomatic infection was estimated to last about three times as long (23 days) as asymptomatic infection (8 days). Note, however, that the prevalence of asymptomatic children was over three times higher than of symptomatic children. The analysis indicates that children with symptomatic infection are no more infectious than those with asymptomatic infection. This seems to contradict experimental work that demonstrated higher levels of shedding in those individuals with more severe infections (Kang *et al*. 2004). This contradiction could be for a number of reasons: the number of asymptomatic infections may be too low to identify this effect; the definition of symptomatic as an infection involving symptoms may be too broad; the removal of children from their DCC due to symptoms could have occurred. Rotavirus reinfection within a child is dependent on the density of infection within his/her DCC with an infection coming from outside the DCC very rarely (table 5, estimates of *λ*_0_).

Previous attempts at investigating the role of acquired immunity (i.e. previous infection) in rotavirus infection have failed to incorporate the transmission dynamic effects included in the model framework used here. In particular, the risk of infection of an individual is determined by both their immune status and their exposure, and not including the latter will result in inaccurate estimates. Unfortunately, information on the first infection, the total number of previous infections and their effect on subsequent infections cannot be obtained since the data are not from a birth cohort. The transmission dynamics of rotavirus repeated infections outside the high prevalence season cannot be explored using the data presented here. The transmission dynamics associated with reinfection, partial and temporary immunity are, generally, highly nonlinear (Gomes *et al*. 2004), and even more so if an effect of dose is included (Gomes *et al*. 2005). Community-based longitudinal data and their appropriate analysis are clearly required if the effect of a given vaccine and vaccination programme is to be understood.

Longitudinal, individual-based data in relatively small populations are increasingly being used to study infection dynamics (Auranen *et al*. 2000, 2004; Eerola *et al*. 2003; Smith & Vounatsou 2003; Basáñez *et al*. 2004; Cooper & Lipsitch 2004; Melegaro *et al*. 2004; Liu *et al*. 2005). Analysis of such data presents several methodological problems. Generally, the estimates of the two processes of incidence and recovery are likely to be highly correlated. If, however, the sampling is dense and testing sufficiently sensitive, then incidence and recovery can be treated separately, as here.

To consider immunity, it is necessary to have information on the history of infection in an individual. The simplest assumption is that an infection ‘resets’ the immune system to maximum. In this case only data from children with multiple infections and only their uninterrupted follow-up since their first measured infection can be used. To consider concepts such as cumulative and cross-immunity, follow-up serological and genotypic data, respectively, from a birth cohort are required.

The maximum measured time since previous infection from our datasets is 119 days and cellular and serological immune responses to rotavirus infection in children have been observed to remain for a few months post infection (Makela *et al*. 2004), thus it should be possible to detect waning effects of previous infection with this dataset. However, the results presented here should be considered within the context of a number of confounding factors. Firstly, the results have not been corrected for age. Secondly, an individual's response to exposure could be dose dependent (if they experience multiple exposures over a short time period or are exposed to a high initial dose) and, since rotavirus is seasonal, responses at the end of the season could be different from those earlier. This effect would be correlated with time since infection. Thirdly, the sensitivity of the diagnostic test for the presence of rotavirus is not 100% and therefore there are a number of choices for the definition of a continued infection.

Time since previous infection, *τ*, was used as a proxy for immunity in our analysis, and the functions *θ*, *f* and *g* of *τ* quantify the risk of symptomatic disease, risk of infection and duration of infection, respectively. The analysis strongly indicates that immunity to rotavirus manifests itself as a waning resistance to disease rather than infection *per se*. That is, if a child has recently recovered from an infection, its next infection will be less likely to involve symptoms if it occurs sooner rather than later. A schematic of partial immunity over time (figure 5) derived from the estimates for models 5 and 8 (table 4) suggests three types of partial immunity developing over different time scales. Immunity to infection (resistance to infection of any kind) is lost within a month and then regained after a few more weeks. This could represent a temporary disruption in protection against disease due to recent infection. Immunity to disease in the form of resistance to symptomatic infection is gained within a couple of weeks and is still waning after three months. Immunity to disease in the form of altered duration of infection is still being gained after two months. A challenge study in pigtailed macaques demonstrated differing profiles for IgA, IgG and IgM antibodies over time since challenge (Westerman *et al*. 2005). Although this occurs over a period of days rather than weeks in animals rather than humans, it does provide evidence that immune responses evolve over different time scales *in vivo* in response to challenge.

The third plot of figure 5 (dashed line) indicates the unexpected result that the duration of infection decreases with time since previous infection. That is, higher immunity correlates with longer durations of infection. This is a counter intuitive result that has also been reported indirectly for malaria, where duration of infection was positively correlated with age (a widely accepted correlate of immunity for this disease; Smith & Vounatsou 2003). A simple exploration of the data implied that right censoring is not responsible for this result. If a series of repeated measures for an individual child ended in a positive value, this infection (a series of consecutive positive values) was excluded from the dataset. An identical analysis performed on this reduced dataset gave similar results. A possible explanation (as an alternative to the long-term development of some sort of immunity against disease) for the result is that the sensitivity of the test combined with heterogeneous shedding has resulted in continued infections being defined as series of repeated infections. Richardson *et al*. (1998) observed continued hospital infections of rotavirus which included consecutive negative samples. If this behaviour also occurs for non-severe and asymptomatic infections, a series of shedding events of decreasing duration would be observed—a profile that would correspond to the third profile of figure 5. Another explanation for this result could be that children who are more susceptible (and therefore become reinfected shortly after recovery) also experience longer infections; however, plots of child-level transmission against time since infection and duration of infection did not support this notion.

The sensitivity of detection of rotavirus infection is a potentially significant confounding factor for the parameter estimation process. The definition of continued infection being a series of samples that end with two sequential negative samples has been used before (see Mohan & Haque 2003). If it is assumed that a negative result is indeed positive when sandwiched between two positive results within a given time period, the profile of past infection is changed for that child. For the case where additional data on the existence of current or recent infection such as antibody level are available, the use of data augmentation approaches (Eerola *et al*. 2003) can be used to estimate the extent of and account for measurement error. For example antibody level rather than time since previous infection could be used as a measure of immunity level within the individual.

The analysis of follow-up data results in different profiles for the natural history of rotavirus reinfection (depending on the definition of a continued infection). The estimates of the transmission coefficient and the average duration of infection are robust to alternative assumptions on the nature of immunity. However, there is very high variation in these values and larger numbers of measurements per child would be necessary to determine the contribution of variation at the child level. The results suggest that there may be some protection against symptomatic infection, and that symptomatic infections have a longer duration than asymptomatic infections. It is possible that we are observing only minor changes in the immune response due to reinfection, when the most significant change would have occurred during the first infection. Only a birth cohort follow-up study would provide the necessary data to test this hypothesis.

These results are of particular interest in view of the likely introduction of live attenuated rotavirus vaccines in many developed countries where DCCs are prevalent. Two candidate vaccines have completed large-scale safety and efficacy clinical trials (Rotarix, GSK Vaccines, and Rotateq, Merck Vaccines), with good safety and efficacy profiles in industrialized countries and in Latin America (Ruiz-Palacios *et al*. 2006; Vesikari *et al*. 2006; Grimwood & Buttery 2007; World Health Organization 2007). Like natural infection, vaccines do not protect against infection but do protect against disease, with repeated rotavirus exposures probable for most children post-vaccination. If the transmission dynamics of wild-type rotavirus will be influenced by the vaccine introduction, especially if a mixed immunized and non-immunized cohort exists in a high-transmission environment such as a DCC, is unclear. A further confounder is likely to be horizontal transmission of vaccine virus, documented at least in previous rotavirus vaccine trials (Jiang *et al*. 2002). It remains likely that the success of rotavirus vaccine at an individual host and population level relies upon the regular re-exposure of vaccine recipients to asymptomatic infection to maintain immunity, at least in the early years.

## Figures and Tables

**Figure 1:**
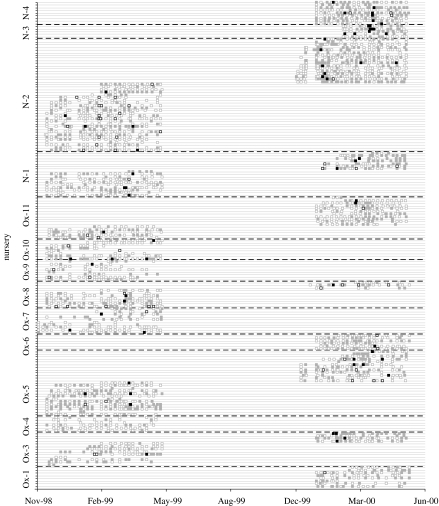
Timeline of samples from children within 14 DCCs. Open and closed squares represent negative and positive samples, respectively. Black and grey squares represent samples taken in the presence and absence of symptoms, respectively.

**Figure 2:**
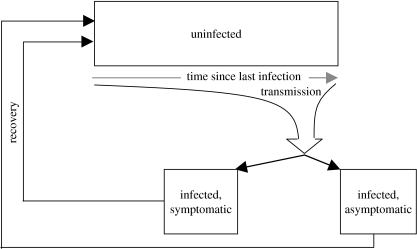
Schematic of the combined parent incidence and recovery models.

**Figure 3:**
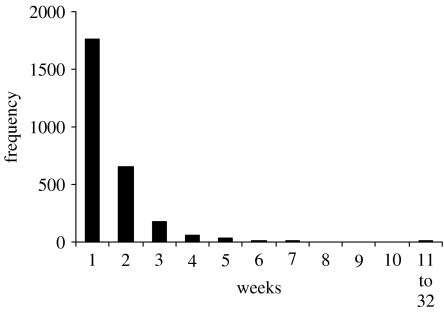
The frequency distribution of intersampling period for the study from which the data are derived.

**Figure 4:**
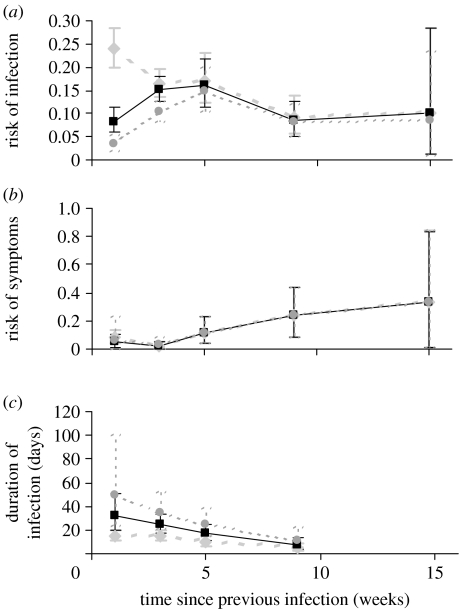
(*a*) The variation of risk of infection, (*b*) risk of disease and (*c*) duration of infection with time since previous infection for *fn0-data* (diamonds), *fn7-data* (squares) and *fn14-data* (circles).

**Figure 5:**
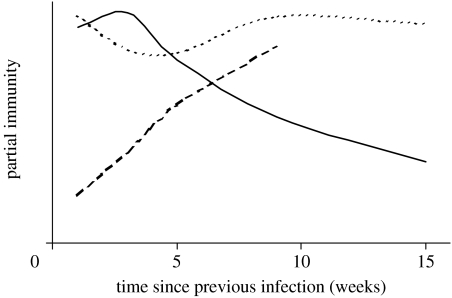
A schematic of development of three types of partial immunity (resistance to infection (dotted line), resistance to symptoms (solid line) and resistance to duration of infection (dashed line)) to rotavirus over time since recovery from a previous infection. This is a redrawing of figure 4 to demonstrate the way each type of immunity varies over time since previous infection.

**Table 1:** A summary of the incidence and recovery models.

	model	definition
incidence	1	force of infection dependent on nursery prevalence
	2	force of infection dependent on nursery prevalence where symptomatic and asymptomatic infections have different contributions
	3	force of infection dependent on nursery prevalence and time since previous infection (categorical)
	4	force of infection dependent on nursery prevalence. Risk of symptomatic infection dependent on time since previous infection (categorical)
	5	force of infection dependent on nursery prevalence and time since previous infection (categorical). Risk of symptomatic infection dependent on time since previous infection (categorical)
recovery	6	constant average duration of infection
	7	different constant average durations of symptomatic and asymptomatic infection
	8	durations of symptomatic and asymptomatic infection dependent on time since previous infection

**Table 2:** A summary of the symbols and definitions of the models.

symbol	definition
*s*_*ijk*_	sample *j* of child *i* in DCC *k* (binary)
*t*_*ijk*_	time since beginning of the study of sample *j* of child *i* in DCC *k* (continuous)
*δt*_*ijk*_	time since previous sample of sample *j* of child *i* in DCC *k* (continuous)
*τ*_*ijk*_	time since previous infection of sample *j* of child *i* in DCC *k* (categorical)
*y*_*d*_(*t*_*ij*_,*k*)	proportion of DCC *k* with symptomatic infection at time *t*_*ijk*_ (continuous)
*y*_*nd*_(*t*_*ij*_,*k*)	proportion of DCC *k* with asymptomatic infection at time *t*_*ijk*_ (continuous)
*m*_*ijk*_	function for estimating the risk of symptoms during an infection
*I*_*ijk*_	indicator variable for an incidence at sample *j* of child *i* in DCC *k* (binary)
*R*_*ijk*_	indicator variable for a recovery at sample *j* of child *i* in DCC *k* (binary)
*f*(*τ*_*ijk*_)	function describing risk of infection over *τ*_*ijk*_
*λ*_*ijk*_	force of infection acting on child *i* at time *t*_*ijk*_
*g*(*τ*_*ijk*_)	function describing altered duration of infection over *τ*_*ijk*_
*δ*_*ijk*_	duration of infection of child *i* at time *t*_*ijk*_
*β*	transmission coefficient
*η*	altered infectiousness of symptomatic versus asymptomatic infection
*λ*_0_	non-DCC force of infection
*θ*(*τ*_*ijk*_)	function describing risk of symptoms over *τ*_*ijk*_
*ρ*	altered duration of symptomatic versus asymptomatic infection

**Table 3:** Transition indicator variables. (The values of *M* denote the fact that the transition is impossible for the given value of *s*_*i*(*j*−1)__*k*_. For example, if *s*_*i*(*j*−1)*k*_=0 then it is impossible for child *i* to recover at time *t*_*ijk*_ since (s)he was not infected at time *t*_*i*(*j*−1)*k*_.)

*s*_*i*(*j*−1)*k*_	*s*_*ijk*_	*I*_*ijk*_	*R*_*ijk*_
0	0	0	*M*
0	1	1	*M*
1	0	*M*	1
1	1	*M*	0

**Table 4:** The number of each type of transition given three different definitions of continued infection.

data set	number of transitions			

	0–0	0–1	1–0	1–1
fn0-data	1013	478	458	503
fn7-data	1013	391	371	678
fn14-data	1013	323	304	813

**Table 5:** Parameter estimates (including 95% CI) and DIC values for each model fit.

	model	parameter	estimate	2.5%	97.5%	DIC
incidence	1	*λ*_0_ (day^−1^)	0.00121	3.08×10^−5^	0.00442	923.1
		*β* (day^−1^)	0.123	0.106	0.141	
		*θ* (-)	0.0536	0.0281	0.0869	
	2	*λ* (day^−1^)	0.00120	3.09×10^−5^	0.00427	923.9
		*β* (day^−1^)	0.134	0.0777	0.209	
		*θ* (-)	0.0535	0.0280	0.0867	
		*η* (-)	0.513	0.204	0.719	
	3	*λ*_0_ (day^−1^)	7.02×10^−4^	1.83×10^−5^	0.00260	910.7
		*β*_1_ (day^−1^)	0.0839	0.0587	0.113	
		*β*_2_ (day^−1^)	0.152	0.1255	0.182	
		*β*_3_ (day^−1^)	0.161	0.1144	0.217	
		*β*_4_ (day^−1^)	0.0856	0.052	0.128	
		*β*_5_ (day^−1^)	0.100	0.01198	0.281	
		*θ* (-)	0.0537	0.02805	0.0866	
	4	*λ*_0_ (day^−1^)	0.00120	3.16×10^−5^	0.00430	917.1
		*β* (day^−1^)	0.123	0.106	0.141	
		*θ*_1_ (-)	0.0529	0.00645	0.142	
		*θ*_2_ (-)	0.0233	0.00493	0.0554	
		*θ*_3_ (-)	0.120	0.0410	0.232	
		*θ*_4_ (-)	0.238	0.0879	0.436	
		*θ*_5_ (-)	0.335	0.0124	0.840	
	5	*λ*_0_ (day^−1^)	6.95×10^−4^	1.77×10^−5^	0.00255	904.7
		*β*_1_ (day^−1^)	0.0836	0.0586	0.113	
		*β*_2_ (day^−1^)	0.153	0.126	0.181	
		*β*_3_ (day^−1^)	0.161	0.114	0.217	
		*β*_4_ (day^−1^)	0.0855	0.0519	0.127	
		*β*_5_ (day^−1^)	0.101	0.0116	0.285	
		*θ*_1_ (-)	0.0525	0.00653	0.142	
		*θ*_2_ (-)	0.0236	0.00503	0.0561	
		*θ*_3_ (-)	0.120	0.0409	0.233	
		*θ*_4_ (-)	0.238	0.0873	0.440	
		*θ*_5_ (-)	0.332	0.0118	0.841	
recovery	6	*δ*_*f*_ (days)	15.3	13.0	17.9	559.2
	7	*δ*_*f*_ (days)	21.1	15.5	27.0	555.6
		*ρ* (-)	0.279	0.118	0.468	
	8	*δ*_1_ (days)	32.2	20.0	50.3	542.6
		*δ*_2_ (days)	24.9	17.7	33.3	
		*δ*_3_ (days)	17.1	11.1	25.2	
		*δ*_4_ (days)	7.33	3.61	14.1	
		*ρ* (-)	0.256	0.101	0.438	
